# Changes in profile of patients seeking alcohol treatment and treatment outcomes following policy changes

**DOI:** 10.1007/s10389-017-0841-0

**Published:** 2017-10-06

**Authors:** Anne-Sophie Schwarz, Bent Nielsen, Anette Søgaard Nielsen

**Affiliations:** Unit of Clinical Alcohol Research, Clinical Institute, University of Southern Denmark, J.B. Winsløws Vej 20, Indgang 220B, 5000 Odense, Denmark

**Keywords:** Alcohol use disorder, Alcohol problems, Alcohol treatment, National register

## Abstract

**Aim:**

In 2007, the legal obligation to deliver alcohol treatment to the public was transferred to the 98 municipalities of Denmark. This resulted in changes in how alcohol treatment centers in Denmark work. The aim of the present study was to describe the patient profiles and treatment outcomes in the alcohol treatment centers regarding regional variation and changes over time.

**Subjects and methods:**

This is a descriptive, register-based study of patients enrolled in alcohol treatment centers from 2006–2014 in Denmark. Only patients above the age of 15 years and with a valid postal code were included. The sample was restricted to the patients’ first contact with the alcohol treatment register (*n* = 44,516).

**Results:**

Patients who initiated treatment in the period 2006–2014 and were registered in the National Alcohol Treatment Register were primarily males (69–70%) with an average age between 46 and 49 years. Most had a vocational education (38–41%) on top of their primary education. The number of years with excessive alcohol use started out as being quite different in the five regions, but became more homogeneous over the study period. Treatment duration in the various regions followed a similar pattern, with all five of them having a similar treatment duration time of 160–230 days by 2014.

**Conclusion:**

We found that treatment for alcohol use disorder became more homogeneous across the regions in Denmark over time and that by 2014 it was difficult to identify any differences across the country.

## Introduction

Alcohol has been identified by multi-criteria decision analysis to be the overall most harmful drug, with heroin and cocaine in second and third place (Nutt et al. 2010). The World Health Organization (WHO) has reported excessive alcohol consumption to be the second most important lifestyle factor (after tobacco use) affecting the overall disease burden in high-income countries such as Denmark (World Health Organization 2009). Alcohol has also been found to be a significant cause of non-communicable diseases (Room et al. 2005). In Denmark, consumption of alcohol leads to at least 3000 potentially preventable deaths each year, representing 5% of total annual deaths, and also contributes to many patient contacts with the health care system (Danish Health Authority and Statens Serum Institut 2015).

Potentially harmful drinking occurs in 20% of the Danish population, and an estimated 5% of the population has developed a dependency that jeopardizes their working career and may lead to early death (Gottlieb Hansen et al. 2011). Hence, during the last 20–30 years, the Danish Health Authority has become increasingly concerned about the quality of the treatment for alcohol use disorder available to the population.

By law, treatment for alcohol problems is offered to the Danish population as it is in most other European countries (The Danish Parliament 2016). The public outpatient treatment for alcohol dependence in Denmark is easily accessible, free of charge and open for self-referral, and patients may remain anonymous during treatment if they so wish. For decades, most treatment for alcohol dependence has been offered on an outpatient basis, with individuals being able to choose freely among the publicly funded treatment offers.

Treatment for alcohol use disorder is delivered per the Danish Health law (January 1, 2007), and for decades the treatment was offered by the Danish counties, who were also responsible for delivering health care.

However, a study in 2004, mainly focusing on how services varied according to opening hours, implementation of manualized treatment methods and use of systematic quality control, illustrated how the treatment services in the Danish regions varied significantly among the various counties (Milter et al. 2004). A network of managers of publicly funded alcohol treatment institutions, Alkoholfagligt Forum (AFF), established in 2002, therefore initiated annual joint seminars for all staff members in the treatment institutions, in addition to meetings between the managers, to discuss, inspire and learn from one another about how to implement in particular evidence-based treatment methods and strategies.

Furthermore, in 2006, a treatment of alcohol dependence assessment was performed and published (Danish Centre for Evaluation and Health Technology Assessment and National Board of Health 2006). This was in many respects the first major step in a national process aimed at improving the quality of treatment and increasing the homogeneity of treatment across the country.

In 2006, the publicly funded treatment of alcohol dependence was still managed by the counties, but became the responsibility of the individual Danish municipalities from January 1, 2007, onwards. In addition, a new law guaranteed that the waiting time for treatment should not exceed 14 days (The Danish Parliament 2016). The political argument for this change was that the local governments thereby would be able to coordinate treatment with social services and provide treatment more locally.

The change was implemented as part of a great reform, consolidating 14 former counties into 5 regions with responsibility mainly for the health care sector and reducing the number of municipalities from 271 to 98 (The Danish Parliament 2005). Hence, instead of treatment for alcohol dependence being the responsibility of 14 Danish counties (plus two local governments with the status of counties), alcohol treatment became the responsibility of 98 local governments. As a result, differences became even more profound across the country, with the number of treated patients varying between 0.08 and 8.37 per 1000 inhabitants and the organization of alcohol treatment differing depending on whether it was part of the community health services (Sundhedsforvaltning) or social services (Socialforvalting) within the municipalities or run by private distributers on behalf of the municipalities (Becker et al. 2012).

Following the reform, additional national initiatives were taken to improve the quality of the treatment for alcohol problems and to some extent to prevent the high number of relatively small local treatment institutions leading to reduced quality of treatment instead of increased quality (Danish Health Authority 2010b). The initiatives provided, for instance, advisory material for the municipalities in 2008, describing what treatment for alcohol dependence should include and what treatment institutions should be able to offer (Becker et al. 2008), based on a treatment of alcohol dependence assessment (Danish Centre for Evaluation and Health Technology Assessment and National Board of Health 2006). In addition, training courses for staff employed in public alcohol treatment has been offered by the Danish Health Authorities regularly since 2008, including basic training in evidence-based treatment, cognitive behavioral therapy and motivational interviewing, and family focus in treatment. The training was not only arranged but also funded by the Danish Health Authorities and freely available for the local governments. The training course was module-based, with five modules for a total of 16 days, and offered on a regular basis several times a year, allowing treatment institutions to dispatch newly trained staff in a timely manner. In addition to the basic 16-day training course, a series of courses in family treatment and training focusing on preventing harm to children in families with alcohol problems were also offered to staff from the alcohol treatment institutions without costs to the local governments (Danish Health Authority 2014).

In 2010, the Danish Health Authority launched a nationwide project for local quality improvement projects in the municipalities, aimed at securing the quality of alcohol treatment and implementing strategies to involve the families (significant others) in the treatment rather than only involving the drinker.

In 2015, the Danish Health Authorities devised and published National Guidelines for the treatment of alcohol dependence (Danish Health Authority 2015) and for treatment of alcohol dependence and concurrent mental illness (Danish Health Authority 2016). Finally, the national parliament decided in 2015 that all alcohol treatment institutions should undergo public supervision on a regular basis.

Several reports (Becker et al. 2012; Milter et al. 2004) have investigated the quality of treatment during the decade that followed public reform and changes in the organization of the alcohol treatment field with a focus on securing the quality of treatment in Denmark. They primarily describe changes in staff composition, treatment offers and collaboration with general practice (Becker et al. 2012). Little, however, is known about whether implementation of the broad national strategies for improving and homogenizing the quality of treatment has, in fact, led to more uniform and effective treatment.

## Aim

The aim of this article was to (1) investigate whether the profile of the patients seeking and receiving alcohol treatment in Denmark has changed during the last decade and (2) investigate whether there were differences in the outcome of the treatment across regions and over time.

## Materials and methods

### Description of the National Alcohol Treatment Register (NAB)

Every person born or living in Denmark receives a unique and secure personal identification number (CPR); coupled with public registers, this provides a comprehensive database on the Danish population from the cradle to the grave. The data are of course protected under the Act on Processing of Personal Data (The Danish Data Protection Agency 2012), and therefore researchers can only access the data after a thorough review process, and the data will not contain the actual personal identification numbers but rather encrypted personal identification numbers. One such public database is the National Alcohol Treatment Register (NAB), where everyone who shows up at public (and to some extent private) alcohol treatment centers is registered and asked a number of questions regarding their alcohol problem (Danish Health Authority 2016). Since 2006, is has been mandatory to log all individuals seeking publicly funded treatment in the Alcohol Treatment Register with information about the individual and the treatment course. The register is aimed purely at planning and research (Danish Health Data Authority 2015). In 2008 and 2010, further variables were added to the register (Danish Health Data Authority 2016).

The NAB contains, among other things, information on alcohol treatment and alcohol consumption as well as several patient socioeconomic variables. The information in the NAB is collected when the patient enters treatment by means of a shortened version of the EuropASI interview (Kokkevi and Hartgers 1995). The NAB started on 1 January 2006, and both public and private alcohol treatment centers are included. It is mandatory to register all publicly funded treatment courses, but private institutions are free to choose whether they register the patient and the treatment course if the patient is paying for treatment his- or herself. However, most treatment courses in Denmark are funded by the local government. Patients who are only receiving medical treatment, being treated by their general practitioner or receiving treatment during incarceration are not registered in the NAB. The reporting is done electronically through the Health Data Protection Agency’s electronic reporting system (SEI) or from the treatment institutions’ own systems directly to the Health Data Protection Agency. Reporting takes places at least once a month, and the register is continuously updated (Danish Health Data Authority 2015).

### Sample

The sample included patients having their first contact with public alcohol treatment centers from 2006–2014.

### Variables

In the study, we included the following variables: whether the patient had any children or not; primary and secondary education (divided into levels); civil status; whether the patient had been in alcohol treatment before or not; whether the patient had received a psychiatric evaluation or not; whether the patient had received Antabuse within the last month or not; the duration of treatment defined as the difference between the admission date and discharge date (where one was given); drinking pattern in the last 6 months; treatment type (out- or in-patient treatment); average weekly alcohol consumption (classified by type of alcohol); end outcome (successful discharge is defined as discharge in agreement with staff or referral to other treatment; unsuccessful discharge is defined as discharge at own request, failure to appear, moved away or deceased and other); referring instance; main source of income; days and years with excessive use of alcohol; and other drug use (cannabis and other drugs) (Danish Health Authority 2010a).

The data used in this article were retrieved through the Danish Health Data Authority, and the project was approved by the Danish Data Protection Agency (Region of Southern Denmark ‘paraplyanmeldelse’ 2008-58-0035 project no. 16/35864).

## Results

A total of 88,057 contacts with the alcohol treatment centers were found from 2006–2014. After cleaning up the data set, 452 observations were removed [299 because of an invalid municipal code, 223 because of invalid age (below 15 or above 100) and 106 empty registrations]. After narrowing the data set to the patients’ first contact with the alcohol treatment centers only, 44,516 observations were left.

### Population characteristics

As can be seen in Table 1, patients who initiated treatment from 2006–2014 and were registered in the National Alcohol Treatment Register were primarily males (69–70%) with an average age between 46 (North Denmark and Central Denmark Regions) and 49 (Capital Region of Denmark) years. Most had a vocational education (38–41%) on top of their primary education. In the Capital Region of Denmark, 10% of the sample had a long-cycle higher education in contrast to approximately 5% for the other regions. Compared to the general Danish population, the numbers are 20% for the Capital Region of Denmark, whereas it is 7–10% in the other regions, differences that are also reflected in our sample. At any given time a total of approximately 15,000 persons annually were receiving alcohol treatment in Denmark during the period.

**Table 1:** Characteristics of persons in alcohol treatment in Denmark

n (%)	Capital Region of Denmark (*n* = 15,384)	Region Zealand (*n* = 5706)	Region of Southern Denmark (*n* = 12,609)	North Denmark Region (*n* = 2999)	Central Denmark Region (*n* = 7818)	All of Denmark (*n* = 44,516)
Children						
Yes	8167 (60)	3735 (69)	6326 (69)	1687 (65)	5036 (70)	24,951 (66)
Primary education						
Still in school	38 (0)	16 (0)	45 (0)	5 (0)	15 (0)	119 (0)
7 years or less	890 (7)	393 (7)	871 (8)	188 (7)	511 (7)	2853 (7)
8–9 years	3725 (28)	2113 (40)	3675 (33)	1038 (39)	2340 (32)	12,891 (33)
10–11 years	4700 (33)	1718 (33)	4230 (38)	942 (35)	2756 (37)	14,346 (36)
High school or equivalent	3777 (28)	962 (18)	1777 (16)	434 (16)	1626 (22)	8576 (23)
Other	203 (2)	57 (1)	629 (6)	77 (3)	151 (2)	1117 (4)
Secondary education						
None	3440 (25)	1483 (28)	3537 (31)	913 (34)	2210 (30)	11,583 (29)
Vocational education (1–4 years)	5096 (38)	2133 (40)	4768 (42)	1117 (41)	2921 (40)	16,035 (40)
Short-cycle higher education (≤3 years)	1280 (9)	545 (10)	1088 (10)	207 (8)	651 (9)	3771 (9)
Medium-cycle higher education (3–4 years)	2351 (17)	841 (16)	1383 (12)	362 (13)	1191 (16)	6128 (16)
Long-cycle higher education (≥4 years)	1362 (10)	291 (5)	540 (5)	111 (4)	395 (5)	2699 (8)
Civil status						
Married	5297 (38)	2360 (44)	4940 (42)	1110 (41)	3142 (42)	16,849 (41)
Divorced/separated	5482 (39)	2047 (38)	4559 (39)	1064 (39)	2770 (37)	15,922 (39)
Widowed	527 (4)	207 (4)	362 (3)	86 (3)	247 (3)	1429 (3)
Never married	2688 (19)	747 (14)	1828 (16)	438 (16)	1295 (17)	6996 (17)
Treated before						
No	6665 (47)	2767 (51)	5673 (60)	1732 (62)	4398 (59)	21,235 (54)
Psychiatric treatment						
Yes	740 (5)	136 (2)	68 (1)	80 (3)	147 (2)	1171 (4)
Received Antabuse						
Yes	4019 (26)	1868 (33)	2740 (22)	1104 (37)	2669 (34)	12,400 (29)
Duration of treatment						
Days	276	285	194	163	286	248
Drinking pattern (last 6 months)						
Every day	6317 (45)	2336 (45)	4151 (44)	1080 (40)	2730 (37)	16,614 (43)
Several days a week	3171 (23)	1284 (25)	2117 (23)	682 (26)	2066 (28)	9320 (24)
Mainly on weekends	780 (6)	315 (6)	671 (7)	184 (7)	624 (9)	2574 (7)
Binge drinking	1529 (11)	462 (9)	959 (10)	279 (10)	739 (10)	3968 (10)
A few days of drinking	1131 (8)	375 (7)	812 (9)	222 (8)	581 (8)	3121 (8)
Occasional 1-day drinking	499 (4)	212 (4)	363 (4)	104 (4)	349 (5)	1527 (4)
Completely sober^a^	463 (3)	228 (4)	294 (3)	118 (4)	193 (3)	1296 (3)
Treatment type						
Out-patient	13,215 (86)	5335 (93)	10,663 (85)	1911 (64)	7327 (94)	38,451 (87)
In-patient	2156 (14)	371 (7)	1946 (15)	1088 (36)	434 (6)	5995 (17)
Average weekly alcohol consumption in units						
Beer	38	36	29	43	31	36
Wine	17	15	11	14	13	14
Spirits	18	15	11	14	12	14
Alcopops	0	0	0	16	0	2
End outcome						
Discharged in agreement with staff	2754 (19)	1295 (24)	4102 (33)	817 (29)	2210 (32)	11,178 (28)
Discharged at own request	1756 (12)	767 (14)	2375 (19)	610 (21)	1257 (18)	6765 (17)
Failed to appear	4706 (32)	1633 (30)	4103 (33)	502 (18)	1856 (27)	12,800 (31)
Moved away or deceased	233 (2)	114 (2)	233 (2)	46 (2)	280 (4)	906 (2)
Other treatment	1267 (9)	247 (5)	800 (7)	212 (7)	318 (5)	2844 (7)
Other	3776 (26)	1309 (24)	688 (6)	668 (23)	1060 (15)	7501 (22)
Referral						
Self-referral	8016 (54)	2861 (51)	6515 (52)	1465 (52)	3473 (43)	22,330 (51)
Social services	1318 (9)	503 (9)	1224 (10)	332 (12)	696 (9)	4073 (9)
Psychiatric and somatic hospitals	2382 (16)	720 (13)	2172 (17)	309 (11)	1187 (15)	6770 (16)
Police	561 (4)	361 (6)	602 (5)	132 (5)	579 (7)	2235 (5)
Civilians	1844 (12)	821 (15)	1451 (12)	438 (15)	1090 (14)	5644 (13)
Other	441 (3)	184 (3)	437 (3)	93 (3)	505 (7)	1660 (4)
Primary source of income						
Earnings	5310 (37)	1896 (35)	5161 (43)	791 (28)	2549 (33)	15,707 (38)
State education grant or rehabilitation benefits	325 (2)	84 (2)	269 (2)	49 (2)	207 (3)	934 (2)
Public benefits	5058 (35)	2016 (37)	3986 (33)	1304 (46)	2821 (37)	15,185 (36)
Early retirement	2871 (20)	1210 (22)	2093 (17)	568 (20)	1525 (20)	8267 (20)
Other	818 (6)	272 (5)	579 (5)	97 (3)	527 (7)	2293 (6)
Excessive use of alcohol						
Days in the last month	17	15	13	13	13	14
Total number of years	14	13	10	12	12	12
Drugs used in the last month						
Cannabis	1438 (9)	333 (6)	648 (5)	117 (4)	593 (8)	3129 (8)
Other drugs	36 (0)	4 (0)	19 (0)	5 (0)	58 (1)	122 (0)

Danish Health Authority (2010a) Fællesindhold for registering af alkoholmisbrugere i behandling [Danish]

^a^Person has completed a treatment course but returns because of a desire for support in staying abstinent, for example. (Danish Health Authority 2010a)

There were more patients below the age of 36 in the North Denmark Region and the Central Denmark Region compared with other regions. The number of patients aged 36–45 years was approximately the same throughout the country, but in the Capital Region of Denmark, the Region of Southern Denmark, and the Region Zealand there were more patients aged 56 years or older compared to other regions.

Most of the patients who initiated treatment were either married (38–44%) or divorced/separated (37–39%), and most had children (60–70%); 37–45% had been drinking every day prior to the start of treatment.

Overall, beer was the preferred kind of alcohol consumed by the patients (29–43 units per week). Spirits and wine were the second most frequently consumed for patients in all regions, except for the North Denmark Region, where alcopops were the second most consumed beverage (16 units per week on average). Curiously, the North Denmark Region was the only region where patients consumed alcopops.

**Fig. 1:**
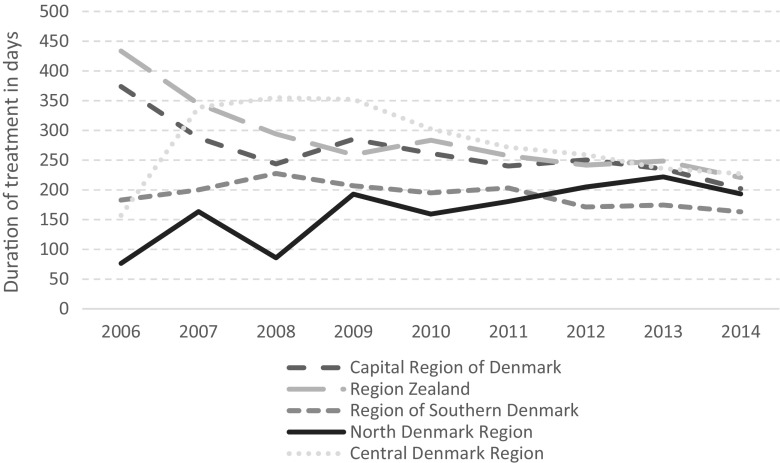
Duration of treatment in days across regions and time

Over time, the duration of treatment courses changed across the regions. In 2006, treatment courses in Region Zealand had the longest duration (433 days on average), and treatment courses in North Denmark had the shortest duration (76 days on average). However, over the period investigated the regions became more homogeneous, with all five regions having a treatment duration of between 160 and 230 days by 2014 (see Fig. 1). During the same period, treatment lengths decreased for all five regions. In the Central Denmark Region, it went from 231 to 168 days between 2006 and 2014, for example.

**Fig. 2:**
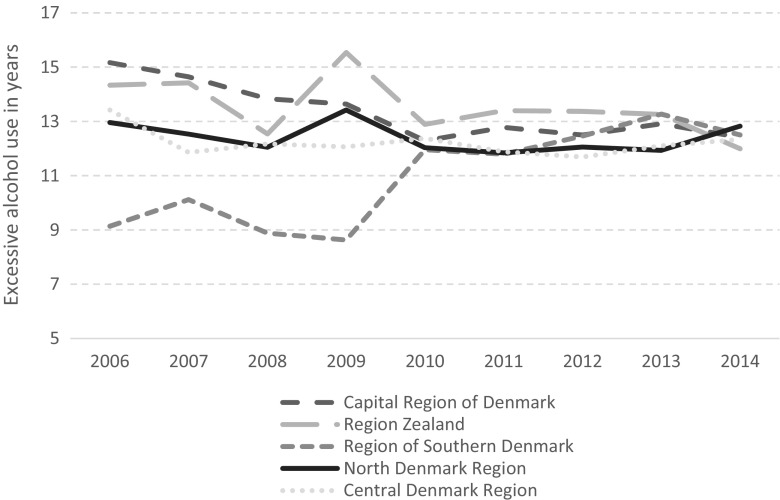
Excessive alcohol use in years across regions and time

A similar picture arose for patient factors. At the beginning of the period under investigation, the patients who initiated treatment in the Region of Southern Denmark had been drinking excessively for 9 years compared to patients in the Capital Region of Denmark, who had been drinking excessively for 15 years. However, over time these differences disappeared between the five regions, with the average number of years of excessive alcohol use stabilizing at 12–13 years across the country (see Fig. 2).

**Fig. 3:**
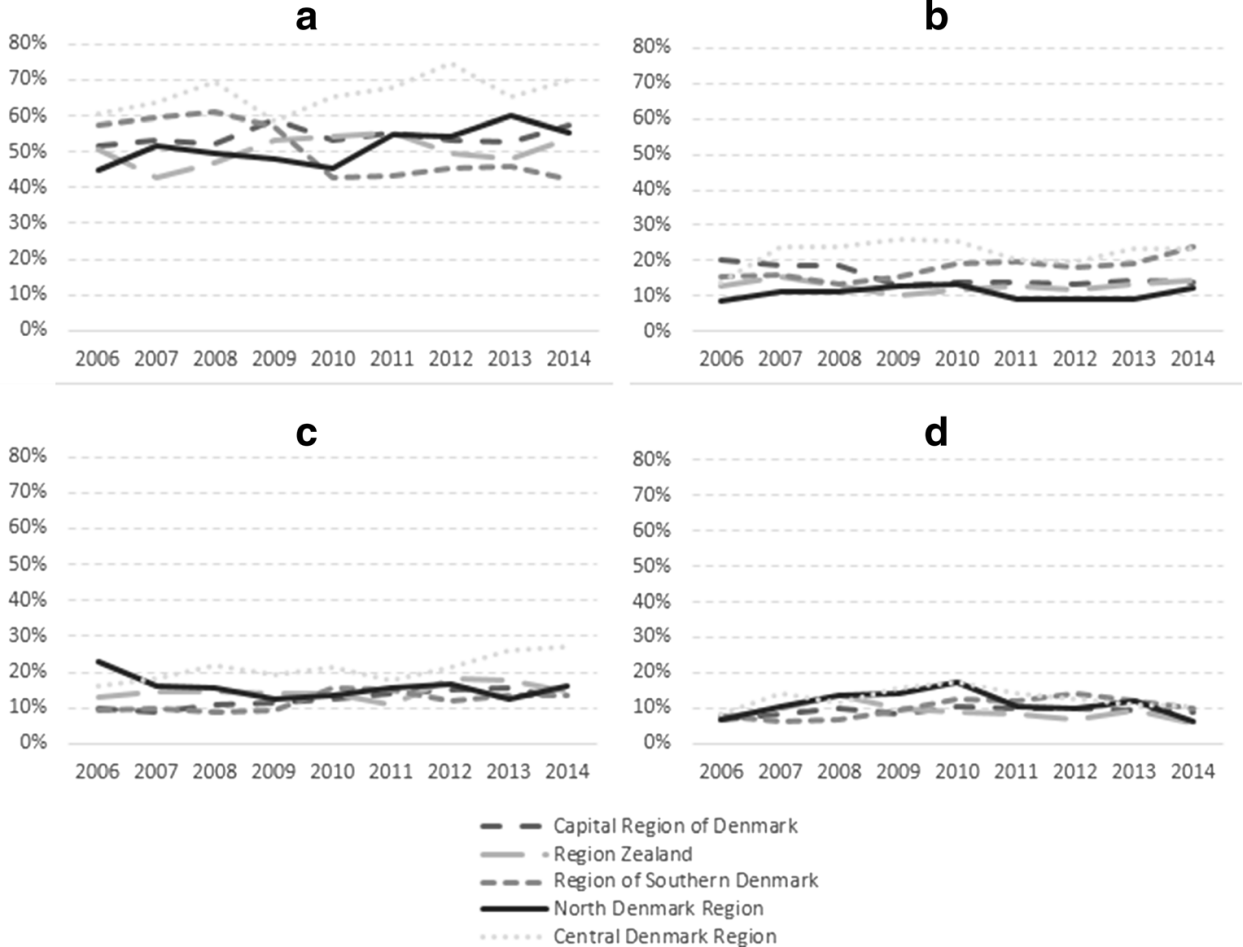
Patient referral from **a** self-referral; **b** psychiatric or somatic hospital; **c** friends or family; **d** social services

Most of the patients (50%) across the country had themselves sought out treatment for their alcohol use disorders, and this percentage was stable across time in the five regions, although differences between the regions did arise over time (see Fig. 3). In 2014, Central Denmark showed the highest percentage of self-referrals at 70%, and the Region of Southern Denmark had the lowest number of self-referrals at 43%. Around 20% of the patients were referred from psychiatric or somatic hospitals, and roughly 10% were referred by social services, with these percentages being similar across the country. Large differences were found in referrals from friends and family, whereby 27% in the Central Denmark Region were referred by friends and family, and only 13% were referred this way in the Capital Region of Denmark and the Region of Southern Denmark.

**Fig. 4:**
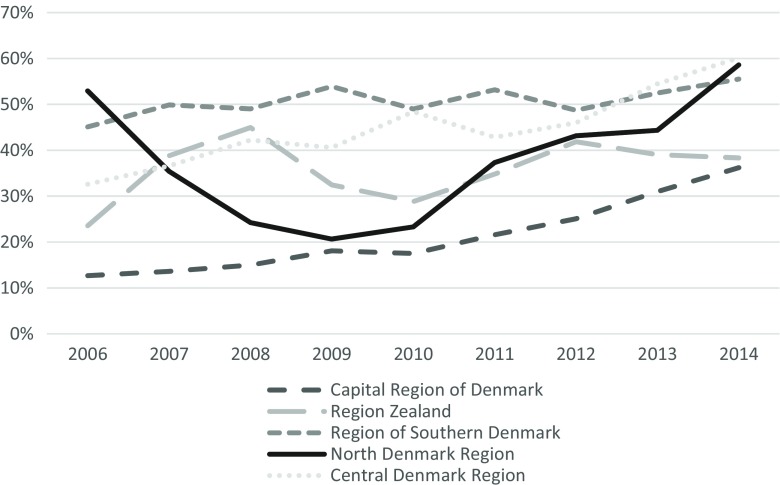
Percentage of successful discharges from alcohol treatment centers

When looking at successful and unsuccessful discharges, there seemed to be an overall trend with the number of successful discharges increasing over time and unsuccessful discharges decreasing over time in most of the five regions (see Fig. 4). Furthermore, a more homogeneous picture also formed over time. Nonetheless, in the Region Zealand and the Capital Region of Denmark, less than 50% of discharges were successful in 2014.

## Discussion

We found that treatment for alcohol use disorder seems to have become more homogeneous across the regions in Denmark over time, and by 2014 it was difficult to identify differences across the country. This increased homogeneity was seen in both relation to the mean length of treatment courses and the number of treatment courses that were concluded in agreement between staff and patient. This may be due to the strong focus on quality assurance and development and series of initiatives from the network of managers (AFF) in general and the Danish Health Authority in particular. In the 1990s there was a rather strong tradition in Denmark for very long, outpatient treatment courses, and this seems to have changed as well. This may be due to an increased common understanding in Denmark of alcohol treatment as something that not only begins, but also has a planned conclusion compared to being a permanent offer.

Many alcohol treatment institutions became part of social services in the municipalities because of the reform in 2006; hence, we had expected the number of referrals to treatment for alcohol use disorder from social services to increase with the reform, but this did not seem to be the case. The pattern of referrals was instead rather stable throughout the investigatory period.

The limitations of the present study relate primarily to the National Alcohol Treatment Register itself (NAB). The NAB is relatively new (from 2006), and especially in the first few years there were registration problems in the different regions in Denmark. For instance, the ‘other’ category for the question of the end outcome was initially used more heavily especially in the North Denmark Region, but this picture changed after a few years.

Another weakness is that when the patients arrive at the alcohol treatment centers, they can choose to remain anonymous. However, only very few patients do so. Also, the majority of the variables in the NAB require mandatory reporting from the alcohol treatment centers, but some variables are not; therefore, these variables are less reliable to look at as they are not consistently reported.

There are of course also strengths of the study. The registration problems that were present in the first years of the register seem to have stabilized. The NAB is a very comprehensive register covering a large amount of information that the patients themselves report. The personal identification number makes it possible to link the information from the NAB to other registers, thus facilitating larger studies involving more registers in the future.

## Conclusion

The profiles of the patients do not vary across the country and hardly vary over time. Thus, the many years with a strong focus on and initiatives within the alcohol treatment field seem to have resulted in largely homogeneous treatment offers across the country.
